# Poverty in the Midst of Plenty: Unmet Needs and Distribution of Health Care Resources in South Korea

**DOI:** 10.1371/journal.pone.0051004

**Published:** 2012-11-30

**Authors:** Jongho Heo, Juwhan Oh, Jukyung Kim, Manwoo Lee, Jin-seok Lee, Soonman Kwon, S. V. Subramanian, Ichiro Kawachi

**Affiliations:** 1 Public Health Joint Doctoral Program, University of California, San Diego and San Diego State University, San Diego, California, United States of America; 2 Institute of Health Policy and Management, Medical Research Center, Seoul National University, Seoul, South Korea; 3 Health, Welfare, Family and Gender Equity Team, National Assembly Research Service, Seoul, South Korea; 4 School of Public Health, Seoul National University, Seoul, South Korea; 5 Department of Society, Human Development, and Health, Harvard School of Public Health, Boston, Massachusetts, United States of America; Tehran University of Medical Sciences, Islamic Republic of Iran

## Abstract

**Background:**

The unmet needs for health care have been used as an alternative measurement to monitor equity in health services. We sought to examine contextual influences on unmet needs for health care whereas precedent studies have been focused on individual characteristics on them.

**Methods and Findings:**

The current study conducted multilevel logistic regression analysis to assess the effects of individual- and contextual-level predictors in meeting individual health care needs in South Korea. We sampled 7,200 individuals over the age of 19 in the Fourth Korea National Health and Nutrition Examination Survey in 2009. Included in the regression model were individual predictors such as demographic variables, socio-economic status, and self-rated health; the density of beds and physicians in public and private sectors within different regions were used as contextual-level predictors. This study showed the inverse association between unmet needs and regional resources in private sectors after controlling for the effects of individual-level predictors.

**Conclusion:**

Our findings suggest that increasing regional resources in private sectors might produce inefficiency in the health care system and inequity in access to health services, particularly where the competition in private health care sectors was highly stimulated under the fee-for-service reimbursement scheme. Policies for the reallocation of health care resources and for reduction of individual health care costs are needed in Korea.

## Introduction

The performance of health systems is a fundamental determinant of population health. Many countries pay particular attention to achieving equity in the health care delivery system. To monitor equity in health services, a body of research used conventional utilization-based indicators; however, the indicators were criticized in that medical utilization may occur independent of need [1], [2]. As an alternative approach, direct measurement of unmet needs for health care has been used to complement the limitation of the utilization-based indicators. Stenvens et al. (1998) defined health care need as “the capacity to benefit from healthcare”; unmet needs occur where patients/individuals remain “non­recipients of beneficial healthcare interventions” [3].

In the last decade, measuring unmet needs for healthcare has been widely used to assess horizontal equity at both local and national levels [4], [5], [6], [7], [8], [9]. Studies have primarily focused on individual-level characteristics as influential factors on the unmet needs for health care [10], [11], [12], [13], [14], [15], [16], [17]. Whereas, there have been a few studies of unmet health needs focused on community-level variations, these studies were largely ecological and did not control for individual-level characteristics. The findings of the studies thus may be a reflection of the composition of communities, rather than an indication that the characteristics of the community themselves influence health care utilization. Examining the effects at the individual- and community-level simultaneously is enabled by multilevel analysis which is a statistically proper method to analyze hierarchically nested data structure.

Several studies have examined the relationship between individuals’ unmet needs for health care and community-level factors with the multilevel method. The studies suggested that regional low socio-economic status (SES) such as high rates of poverty and unemployment [18], [19], low levels of educational attainment within communities [18], [19], [20], social disruption [21], social capital [21], and the degree of urbanization [18], [21] may influence health status and hence the need for health care. In contrast to studies which focused on consumers’ utilization, some studies have examined supply-side factors (e.g. primary care physicians, dentists, psychiatrists) [18] or hospital beds [19], [21] and showed that they do not necessarily correlate with the level of unmet needs.

In South Korea, as in other countries, there are persistent indications of unmet needs in spite of the achievement of universal health care coverage in 1989. The studies in Korea have identified high levels of out-of-pocket payments in health care expenditure as a major cause of unmet needs in universal health care coverage [22], [23] and suggested improving limited benefit coverage to enable easier access to health care [7], [23], [24], [25]. On the other side, there was no ecological research to elucidate the effect of contextual level factors on regional unmet needs for health care in Korea. To our knowledge, there also has been no study to identify the individual- and contextual-level predictors of unmet needs for South Korean health care via multilevel analysis.

We sought to conduct a study to examine individual- and contextual-level predictors of unmet needs for health care in Korea by multilevel analysis. We hypothesize that contextual-level predictors may enable or impede the ability of individuals to meet their health care needs.

## Methods

### Source of Data

The nationally representative cross-sectional samples of 10,533 individuals were derived from The Fourth Korea National Health and Nutrition Examination Survey (KNHANES IV-3), 2009, conducted by the Korea Centers for Disease Control and Prevention (KCDC). The KNHANES IV was approved by the ethics committees of the KCDC. The survey consisted of three components: health interview surveys, health examination surveys, and nutrition surveys of a representative sample of the non-institutionalized civilian population aged 1 year or older in South Korea. The survey employed three stage stratified multistage probability sampling based on geographical area, gender, and age of household registries of the 2005 National Census Registry. The response rate of the KNHANES IV-3 was 79.2% (N = 10,078) of the total 12,722 sample pool. We selected adults over the age of nineteen and excluded observations which had missing data as they were less than 5% of all respondents. The final national sample included 7,200 individuals from 16 metropolitan areas and provinces which cover the entire country. As region-level variables, density of health care resources in public and private sectors were provided by the Korean Health Insurance Review and Assessment.

### Definition of Variables

#### Outcome variables

There have been two approaches to measure unmet needs for health care: clinical and subjective [6]. The former assesses whether individuals received appropriate care based on clinical guideline [26]. The latter relies on individuals’ self reports of whether they received the care they needed based on their own perceptions. Numerous existing studies have included subjective assessments regarding unmet needs for health care since individuals are better able to estimate their own health status [4] and identify barriers to receiving care. Our outcome measures followed the conventional assessment for subjective health care needs. The measurement of unmet needs for health care was dichotomized based on the KNHANES survey questionnaire: “Was there ever a time when you felt that you needed health care but you didn’t receive it in the past 12 months?” A “yes” response was treated as a definition of an experience of unmet need.

#### Individual level variables

Individual level variables consisted of socio-demographic characteristics including gender, age, income quartile, education, marital status, and self-rated health as well. Age was treated as a continuous variable in the multilevel analysis. We selected the income quartile of individuals to estimate economic status. Education was categorized into four measurements (< = elementary school, middle school, high school, and > = college). Marital status was classified with five categories: never married, married, separated, widowed, and divorced. The measure of self-rated health includes five-categories from very good to very poor.

#### Contextual regional variables

We selected region-level variables reflecting healthcare provision in 2008 and modeled a one-year time lag. We included four regional variables for medical supply: 1) the number of hospital beds per 1,000 residents in public sectors, 2) the number of hospital beds per 1,000 residents in private sectors, 3) the number of physicians per 1,000 residents in public sectors, and 4) the number of physicians per 1,000 residents in private sectors. Public hospitals are those established by the national and regional government authorities, whereas private hospitals are those established by individuals and corporations. We excluded long-term care hospitals and specialized hospitals which provide nationwide health care services such as tuberculosis treatment.

### Statistical Analysis

A two-level multilevel logistic regression model, with individuals at level-1 and 16 metropolitan areas and provinces at level-2, were used to estimate the contribution of contextual and individual determinants simultaneously on unmet needs for health care. The model was fit using multilevel software *MLwiN* (Version 2.22). Random intercept models were built as regional healthcare provisions can vary with respect to the prevalence of unmet needs but not with respect to the effects of the residents’ characteristics. We employed the Markov Chain Monte Carlo (MCMC) function by Bayesian approach to perform the analysis. First, we presented the descriptive data concerning the 16 regions in regard to prevalence of unmet needs and distribution of health care resources. Model 1 assessed the effects of socio-demographic characteristics (gender, age, status of income, education, and marriage, and self-rated health). Then, Model 2 and 3 were analyzed adding contextual variables into Model 1. The models with individual-level characteristics were fit and evaluated how the effect size changed after including predictors of region-level. Lastly, we examined probabilities of unmet needs for health care according to significant regional variables.

## Results

Table 1 shows the prevalence of unmet needs for health care and distributions of health care resources per 1,000 residents across 16 regions. Overall, the private health care resources outweighed the public parts. Health care resources in private sectors took at least 75% out of the total national health care resources (regional range of beds: 74.9- 99.6%; physicians: 76.2%–97.7%). A preliminary analysis showed that there is a trend toward increasing beds in the private sectors as the prevalence of unmet needs rise (*r* = 0.117, *p*<0.0001); however, there is no statistical significance in correlations between the unmet needs and the public sectors.

**Table 1:** Prevalence of unmet needs for health care and distributions of health care resources in 1,000 residents across regions.

		Beds*				Physicians*			
Regions	Unmet needs for healthcare (%)	Public sectors		Private sectors		Public sectors		Private sectors	
Jeonbuk	40.3	1.11	(10.9)	9.11	(89.1)	0.56	(23.8)	1.79	(76.2)
Jeonnam	34.2	1.06	(8.8)	10.98	(91.2)	0.38	(19.6)	1.56	(80.4)
Gwangju	33.3	0.95	(8.5)	10.24	(91.5)	0.43	(16.5)	2.17	(83.5)
Ulsan	31.2	0.03	(0.4)	7.68	(99.6)	0.04	(2.3)	1.70	(97.7)
Gyeongnam	31.0	1.10	(9.5)	10.49	(90.5)	0.34	(19.4)	1.41	(80.6)
Busan	30.2	0.88	(9.2)	8.69	(90.8)	0.21	(8.9)	2.16	(91.1)
Daegu	29.8	0.73	(8.1)	8.27	(91.9)	0.33	(12.8)	2.24	(87.2)
Chungnam	28.9	0.91	(11.6)	6.91	(88.4)	0.28	(14.9)	1.60	(85.1)
Gyeongbuk	27.6	0.68	(7.4)	8.52	(92.6)	0.22	(13.8)	1.37	(86.2)
Daejeon	27.5	1.76	(19.4)	7.30	(80.6)	0.42	(16.5)	2.12	(83.5)
Chungbuk	21.1	0.97	(10.9)	7.94	(89.1)	0.37	(20.3)	1.45	(79.7)
Incheon	20.3	0.43	(5.8)	6.95	(94.2)	0.07	(4.0)	1.70	(96.0)
Jeju	19.9	1.48	(23.2)	4.89	(76.8)	0.36	(19.1)	1.52	(80.9)
Seoul	19.9	0.72	(10.9)	5.90	(89.1)	0.26	(8.2)	2.92	(91.8)
Gyeonggi	19.3	0.66	(9.9)	6.04	(90.1)	0.13	(7.4)	1.63	(92.6)
Gangwon	14.0	2.59	(25.1)	7.71	(74.9)	0.48	(22.6)	1.64	(77.4)

* Number of health care resources per 1,000 residents and percentage of total regional health care resources.

Table 2 shows the distributions of predictors related to unmet needs for health care. Out of a total 7,200 individuals, 1,796 adults (24.9%) experienced unmet needs for health care. Females reported unmet needs at a higher rate than males. Adults who were either young or economically poor experienced more unmet need for health care. The prevalence of unmet need increased with lower levels of educational attainment. Married adults reported the lowest level of unmet needs.

**Table 2:** Prevalence of unmet needs for health care and characteristics of Korean adults.

		Unmet	Met	P-value*
Level 1: Individuals(n = 7,200)		N (%)	N (%)	
Unmet needs for healthcare in this Year	Yes	1,796 (24.9)	–	
	No	–	5,404 (75.1)	
Gender	Male	622 (20.0)	2,495 (80.0)	
	Female	1,174 (28.8)	2,909 (71.2)	<0.001
Age	19–39	630 (27.0)	1,705 (73.0)	
	40–64	772 (23.6)	2,498 (76.4)	
	65+	394 (24.7)	1,201 (75.3)	<0.001
Income Quartile	1st	521 (29.3)	1,257 (70.7)	
	2nd	457 (25.3)	1,350 (74.7)	
	3rd	438 (24.0)	1,384 (76.0)	
	4th	380 (21.2)	1,413 (78.8)	<0.001
Education status	= < elementary school	589 (29.4)	1,417 (70.6)	
	middle school	202 (24.8)	612 (75.2)	
	high school	577 (22.8)	1,953 (77.2)	
	college or higher	428 (23.1)	1,422 (76.9)	<0.001
Marital Status	Never married	268 (25.9)	766 (74.1)	
	Married	1,205 (23.4)	3,955 (76.6)	
	Separated	28 (26.2)	79 (73.8)	
	Widowed	218 (32.5)	452 (67.5)	
	Divorced	77 (33.6)	152 (66.4)	<0.001
Self-rated Health	Very poor	146 (47.4)	162 (52.6)	
	Poor	550 (37.4)	920 (62.6)	
	Moderate	556 (23.1)	1,854 (76.9)	
	Good	499 (18.6)	286 (86.4)	
	Very good	45 (13.6)	286 (86.4)	<0.001
**Level 2: Regions (n = 16)**		**N (%)**	**N (%)**	
Number of hospital beds in public sectors per 1,000residents	<1	1,293 (23.6)	4,283 (76.4)	
	> = 1	503 (29.2)	1,221 (70.8)	<0.001
Number of hospital beds in private sectors per 1,000residents	<7.5	829 (21.1)	3,096 (78.9)	
	> = 7.5	967 (29.5)	2,308 (70.5)	<0.001
Number of physicians in public sectors per 1,000residents	<0.2	424 (20.5)	1,644 (79.5)	
	> = 0.2	1,372 (26.7)	3,760 (73.3)	<0.001
Number of physicians in private sectors per 1,000residents	<2	1,156 (24.8)	3,509 (75.2)	
	> = 2	640 (25.2)	1,895 (74.8)	<0.001

* P-value: Chi-square test about the difference of unmet needs of health care across different socio-demographic groups.

**Figure 1:**
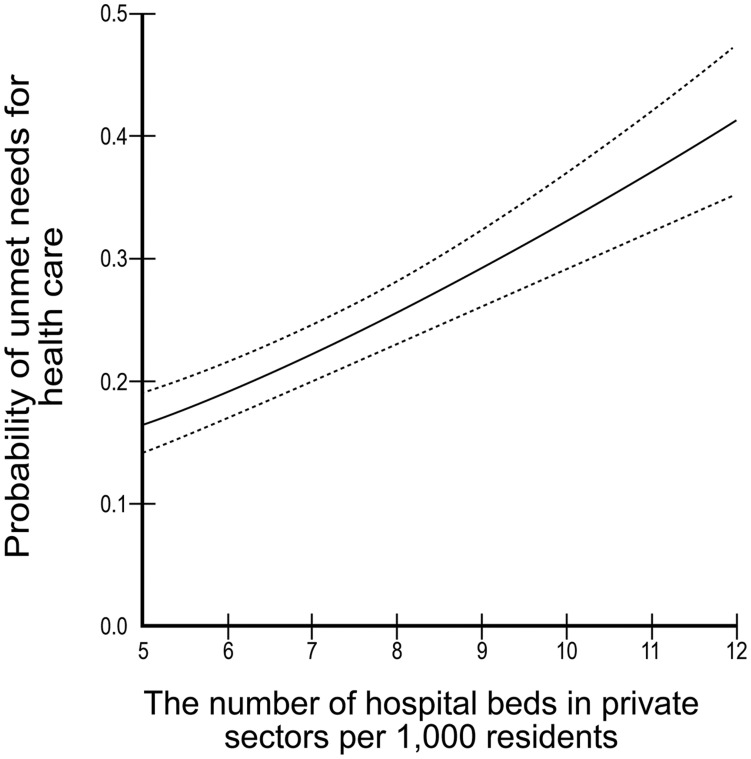
Probability of unmet needs for health care across number of hospital beds in private sectors per 1,000 residents.

Table 3 shows the results of the two-level binomial logit models used to predict the probability of unmet need for health care. The results from the individual characteristics were consistent with the unadjusted results of Table 2. The lower value of -2log(likelihood), the better model; the model 3 is better than others. Of the contextual factors (model 3), the regions that had the highest number of hospital beds in the private sectors were associated with a higher prevalence of unmet needs (Figure 1). There was no statistically significant association between regional distribution of physicians and unmet needs for health care (model 2).

**Table 3:** Multilevel logistic regression estimates (along with their SE) based on two-level binomial logit models for unmet health care need of Korean adults.

	Model 1				Model 2					Model 3				
	Estimate	S.E.	CI		Estimate		S.E.	CI		Estimate		S.E.	CI	
*Fixed Parameters*														
Constant	0.999*	0.183	(0.597 1.329)		1.287*		0.275	(0.885 2.114)		−0.321		0.268	(−0.783 0.208)	
**Individual variables**														
Gender (vs. Male)	0.31*	0.061	(0.192 0.433)		0.316*		0.061	(0.198 0.438)		0.311*		0.062	(0.188 0.428)	
Age	−0.023*	0.003	(−0.029 −0.017)		−0.023*		0.003	(−0.029 −0.018)		−0.023*		0.003	(−0.029 −0.017)	
Income Quartile (vs. 4th)														
1st	0.234*	0.08	(0.074 0.39)		0.236*		0.086	(0.065 0.407)		0.223*		0.087	(0.042 0.385)	
2nd	0.082	0.079	(−0.073 0.235)		0.088		0.084	(−0.074 0.253)		0.077		0.082	(−0.089 0.236)	
3rd	0.072	0.081	(−0.085 0.232)		0.075		0.083	(−0.085 0.239)		0.075		0.087	(−0.095 0.24)	
Education status (vs. college or higher)														
< elementary school	0.311*	0.107	(0.092 0.516)		0.312*		0.108	(0.1 0.526)		0.307*		0.108	(0.077 0.499)	
middle school	0.169	0.113	(−0.067 0.381)		0.174		0.11	(−0.036 0.395)		0.174		0.109	(−0.037 0.378)	
high school	−0.133	0.076	(−0.277 0.018)		−0.14*		0.079	(−0.296 0.013)		−0.136		0.073	(−0.287 0.001)	
Marital Status (vs. Never married)														
Married	0.09	0.096	(−0.096 0.285)		0.088		0.099	(−0.106 0.283)		0.086		0.092	(−0.087 0.265)	
Separated	0.086	0.247	(−0.375 0.564)		0.08		0.243	(−0.416 0.52)		0.07		0.243	(−0.418 0.531)	
Widowed	0.435*	0.15	(0.151 0.718)		0.435*		0.149	(0.138 0.733)		0.438*		0.145	(0.16 0.737)	
Divorced	0.37*	0.176	(0.02 0.715)		0.3798*		0.172	(0.025 0.717)		0.387*		0.173	(0.038 0.713)	
Self Rated Health	−0.455*	0.029	(−0.512 −0.397)		−0.455*		0.03	(−0.511 −0.396)		−0.449*		0.03	(−0.51 −0.387)	
**Contextual variables**														
Number of physicians per 1,000 residents														
In public sectors					0.668		0.712	(−0.912 1.966)						
In private sectors					−0.257		0.189	(−0.69 0.116)						
Number of hospital beds per 1,000 residents														
In public sectors										−0.152		0.12	(−0.384 0.077)	
In private sectors										0.184*		0.024	(0.14 0.238)	
*Random Parameter*														
Region level	0.125	0.061	(0.05 0.275)		0.161		0.09	(0.056 0.39)		0.062		0.038	(0.017 0.155)	
Individual level	1	0	1	1	1		0	1	1	1		0	1	1
-2log(likelihood):	7610.686				7611.259					7610.504				
Units: Regions	16				16					16				
Units: Individuals	7200				7200					7200				

## Discussion

The current study showed an inverse relationship between the number of regional hospital beds per 1,000 residents in private sectors and unmet needs for health care in Korea. Our most notable finding is that–net of individual-level characteristics–the experience of unmet needs was higher in regions with the highest level of private hospital beds per 1,000 residents. This contradicts previous studies, which reported a lack of association between supply of health care resources and unmet health care needs [18], [21] or a significant positive association, if any, with health care supply [19].

This is the first study to report the association between unmet needs and regional resources with regard to health care services in South Korea. The strength of our investigation is the simultaneous consideration of individual characteristics and area-level characteristics within a multi-level analytical framework [27]. Our findings prompt careful consideration of the unique history and evolution of policies which determine the supply of medical resources within the Korean health system. The government, at the introductory stage of health supply policies in 1970–80s, instead of investing in public health care sectors, stimulated the private sectors by providing financial subsidies and administrative supports to meet the people’s health care needs rapidly (at the same time increasing the numbers of medical schools and the entrance quota among private universities) [28], [29], [30]. Spurred utilization of medical services by the national health insurance program introduced in 1977, enabled many physician clinics to have inpatient facilities and hospitals which relied on out-patient services for profit by entrepreneurial physicians [28], [29]. In part due to government policies, the number of acute-care hospital beds per 1,000 capita grew to nearly double the OECD average; however, the number declined in all other OECD countries over time except Turkey [31]. In the 2000s, the private sector dominates the health care system in Korea while the role of the public sector is very limited–96% of hospitals and clinics are privately-owned and they account for 90% of beds while the proportion of public hospital beds are about 10% [32].

Moreover, due to ineffective regulations on private hospitals, the Korean health care delivery system has no mechanism for functional differentiation and referral arrangement across levels of the system [28], [29]. Under the supply overflow and the disorganized health delivery system, competition among private hospitals has intensified [28], [29]. As the revenue of most private hospitals is made up almost exclusively of patient care without philanthropic donations or government subsidy [29], they behave as for-profit entities notwithstanding their not-for-profit status in legal terms [29], [31], [32]. They compete through quality of medical services rather than price under the fee-for-service reimbursement scheme [29], [31], [32]. Competition based on quality translates to expansion of specialists, high-tech equipment, and reduced waiting times. The widening gap of quality medical services among hospitals spurred the excessive expansion of private hospital beds. In short, to win the “medical arms race”, private hospitals have competed to have more beds for more financing. They could easily induce the demand of uninsured services and charge higher prices for them than their public sector counterparts. As a result, the average length of stay is one and a half times longer than the OECD average [31]. Precedent studies from other countries and Korea were consistent with our claim that increased competition leads to high hospital costs [33], [34], [35], [36], [37]. In addition, these inefficient behaviors by the hospitals and the high level of out-of-pocket payment caused by low level of national health care coverage in Korea–amounting to 4.6% of household final consumption in 2007, the third highest in the OECD area [31]–has hindered an individual’s ability to meet their medical needs. Consequently, high competition in the regions where hospital beds in private sectors were overflowed has raised medical costs; thus, it might lead to more unmet needs reports by residents under the low level of national health care coverage.

Private hospitals’ creating needs might be another potential mechanism of the paradoxical result. In regions with higher private sector involvement, there might be more advertising and “want creation”, resulting in increased demand and a perceived need of health services. It might be implied that at least some part of the “unmet” need is actually “unnecessary” need (i.e. excessive and unwarranted demand). As some of the medical services were not affordable to some people in the community, perceptions of unmet need might be higher in communities where more private hospital beds exist. This dynamic has certainly been documented when there is an over-supply of physicians, i.e. lots of unnecessary procedures being carried out under a fee-for-service scheme [29], [31], [32], [33], [38].

Thirdly, the lower quality of medical services of public hospital physicians in regions where there is high competition among private hospitals might enhance unmet needs of the residents. The public hospital physicians who work in these regions may be frustrated, lowering their motivation and think about their job ad hoc or work without full activation [39], [40]. Experiencing relative deprivation from lower salary, insufficient medical resources, and poor circumstances compared to a private hospital, physicians might contribute to public hospital physicians’ lower quality of care, which leads to more unmet needs [39], [40], [41]. This potential strategy may enhance unmet needs which might have been covered via physicians in public hospitals, especially for poor individuals.

Reversely, private hospitals tend to be located and provide more beds where the unmet needs are greatest to respond to commercial opportunities. To exclude this possibility of reverse causality, we intentionally used healthcare provision dataset of one year prior to the KNHANES survey to model a one-year time lag. However, the additional beds provision to meet the unmet needs could be private hospitals’ a reasonable commercial response if the unmet needs in a region have persisted not to decreased.

We found no association between the contextual variables of hospital beds and physicians in public sectors and unmet needs. This may be a result of the exceptional lack of physicians in public sectors, which constitute less than 0.3 physicians per 1,000 populations, one of the lowest in the OECD countries [31]. With regard to individual characteristics, similar to previous work, we found that individuals who reported unmet needs were more socioeconomically disadvantaged than the general population. We confirmed that low income and education, and a marital status of either widowed or divorced were associated with a greater likelihood of reporting an unmet need for health care.

Limitations to this study should be noted. Firstly, the density of hospital beds might not be a representative variable considering growing non-hospital services such as ambulatory care services or same-day surgery. However, there has been no incentive such as global budgeting, diagnosis related group (DRG), which could encourage more same-day surgery in Korea, or to induce adequate medical utilization under the context that high proportion of uncovered treatment and majority of private hospitals behave for-profit. Secondly, the number of hospital beds and physicians per population has been used as representative variables for overall medical resources to correspond to the perceived unmet needs in Korea’s unique medical context [18], [21], [31], [42]. Moreover, we cannot estimate associations between health care provisions in regions and the unmet needs of specific health care services (e.g. in-patient services, out-patient services, mental health services, childcare services, and ambulatory care services) because the KNHANES survey does not ask about the kinds of unmet needs for medical services. Lastly, the technology status, another factor of medical resources, was not included in the analytic models due to the limitations of available data.

Despite the limitations discussed above, multilevel analyses on unmet needs for health care among South Koreans reveal several important insights. The policy for the allocation of health care resources in public sectors should execute specifically to the regions reporting high level of unmet needs in spite of a plethora of health care resources by the private sector in the regions. Policies such as purchasing private hospitals by regional governments and regional regulations for the quota of hospital beds could also be effective. In the long term, efforts to reduce the level of out-of-pocket payment and raise the coverage level of national health care insurance are needed for regional equity for health care access in Korea.
