# Correction: Poverty in the Midst of Plenty: Unmet Needs and Distribution of Health Care Resources in South Korea

**DOI:** 10.1371/annotation/3688fbc5-1878-4752-9cb1-c24486708d17

**Published:** 2012-12-21

**Authors:** Jongho Heo, Juwhan Oh, Jukyung Kim, Manwoo Lee, Jin-seok Lee, Soonman Kwon, S. V. Subramanian, Ichiro Kawachi

The second author's name was spelled incorrectly. The correct name is: Juhwan Oh.

